# Self-Assembly of Colloidal Nanocomposite Hydrogels Using 1D Cellulose Nanocrystals and 2D Exfoliated Organoclay Layers

**DOI:** 10.3390/gels3010011

**Published:** 2017-03-17

**Authors:** Takumi Okamoto, Avinash J. Patil, Tomi Nissinen, Stephen Mann

**Affiliations:** 1 Centre for Organized Matter for Chemistry and Centre for Protolife Research, School of Chemistry, University of Bristol, Bristol BS8 1TS, UK; okamoto.bristol@gmail.com (T.O.); tomi.nissinen84@gmail.com (T.N.); s.mann@bristol.ac.uk (S.M.); 2 Denso Corporation, 1-1, Showa-cho, Kariya, Aichi 448-8661, Japan

**Keywords:** cellulose nanocrystals, organoclay, nanocomposite hydrogels

## Abstract

Stimuli-responsive colloidal nanocomposite hydrogels are prepared by exploiting non-covalent interactions between anionic cellulose nanocrystals and polycationic delaminated sheets of aminopropyl-functionalized magnesium phyllosilicate clays.

## 1. Introduction

Hydrogels have emerged as an important class of soft materials that have found numerous applications ranging from cosmetics and personal care products to biotechnological and biomedical applications. A typical hydrogel comprises a physically or chemically cross-linked 3D network of natural or synthetic building blocks, which has the ability to encapsulate an extremely high percentage of water compared with their dry weight. Significantly, the capacity to retain water and degree of swelling can be regulated by tailoring the cross-linked network of building blocks in the gel matrix. As a result, the physico-chemical (porosity and hydrophilicity) and mechanical (viscoelastic) properties of hydrogels can be modulated for desired applications [1].

Traditionally, hydrogel assembly involves cross-linking of components such as synthetic or natural polymers [2] and biological molecules [3], or self-assembly of low molecular weight gelators [4,5,6,7], or both. Alternatively, recent studies have indicated that self-supporting nanocomposite hydrogels can be prepared by integrating nanoparticles or nanostructures into 3D hydrated cross-linked polymer or biopolymer networks [8,9]. This approach has opened up new opportunities to introduce novel physical, chemical, mechanical, electrical, magnetic, and optical properties into soft materials. For example, materials such as carbon nanotubes and graphene [10,11], polymeric nanoparticles [12,13], inorganic nanoparticles (hydroxyapatite, calcium phosphate, synthetic clays) [14,15,16], and metallic/metal-oxide nanoparticles (gold, silver, iron oxide, titania) [17,18,19,20] have been physically or chemically incorporated within polymeric networks to produce nanocomposite hydrogels with reinforced properties. In this study, we present a new type of stimuli-responsive nanocomposite hydrogel based on the cooperative assembly of two aqueous colloidal sols comprising 1D cellulose nanocrystals (CNCs) and 2D exfoliated sheets of an organically modified magnesium phyllosilicate clay (organoclay). Materials based on CNCs have received considerable attention in recent years due to their applicability in diverse areas such as composites [21], wound dressing and medical implants [22], and as chiral templates for synthesis of inorganic materials [23,24,25]. To the best of our knowledge, we demonstrate the first example of an organic–inorganic hybrid hydrogel in which non-covalent interactions between 1D and 2D nanoparticles spontaneously form a 3D cross-linked matrix, which expedites the entrapment of water to produce a self-supported colloidal nanocomposite hydrogel matrix. Notably, the CNC–organoclay nanocomposite hydrogels can be disassembled and reconstructed by exposing the soft materials to ammonia and carbon dioxide gases, respectively, suggesting that they could be developed as environmentally sensitive soft materials. We also show that the nanocomposite hydrogels are capable of storing and releasing small drug molecules such as ibuprofen. Significantly, introduction of ibuprofen facilitates co-assembly of the organoclay sheets to produce mesolamellar domains within the hydrogel matrix.

## 2. Results and Discussion

**Figure 1:**
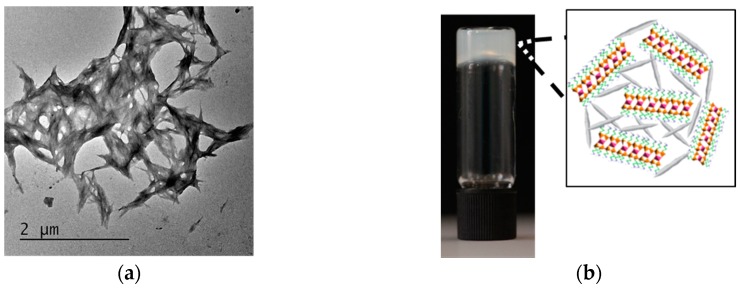
(**a**) Unstained TEM image of cellulose nanocrystal (CNC)-organoclay hydrogel; (**b**) photograph showing self-supported CNC:organoclay (1:0.13) colloidal nanocomposite hydrogel, inset showing schematic illustration of cross-linked network formed by non-covalent interactions between CNC (grey) and exfoliated organoclay sheets.

CNCs were prepared by sulfuric acid hydrolysis of microcrystalline cellulose powder (see Experimental section) [26]. Transmission electron microscopy images of uranyl acetate-stained CNCs revealed the presence of rod-like morphologies, with lengths of 100–300 nm and a thickness of 10–30 nm (Supporting Information, Figure S1a). Aminopropyl-functionalized magnesium phyllosilicate was synthesized using our previously reported studies (see Experimental section) [27]. For delamination, organoclay powders were dispersed in distilled water using ultrasonication. As a consequence, protonation of the aminopropyl-functional groups associated with the inorganic framework facilitated exfoliation of stacks of organoclay sheets and produced a clear suspension containing 50–300 nm sized delaminated organoclay particles (Supporting Information, Figure S1b). CNC–organoclay nanocomposite hydrogels were prepared by adding aqueous dispersions of exfoliated aminopropyl-functionalized magnesium phyllosilicate clay (1–10 wt %) to a 3 wt % colloidal sol of CNCs. The resulting mixtures showed gradual increase in viscosity with increased loadings of the exfoliated organoclay. TEM images of unstained CNC–organoclay hydrogel samples showed the presence of large aggregates comprising cross-linked networks of CNC nanoparticles (Figure 1a). Energy dispersive X-ray (EDX) analysis of the hybrid gel samples showed the presence of Si, Mg, and Cl (counter ion) associated with the organoclay particles and S from sulfonic functional groups of the CNC nanoparticles (Supporting Information, Figure S2). Typically, weight ratios of CNC:organoclay in the range of 1:0.03, 1:0.06. 1:0.13, 1:0.2, and 1:0.26 produced opaque self-supported hydrogels (Figure 1b), and showed no gravity-mediated flow upon inversion of the hydrogels. In contrast, mixing CNC and organoclay dispersions at weight ratios of 1:0.015 and 1:0.033 produced free-flowing liquid and viscous suspensions, respectively.

**Figure 2:**
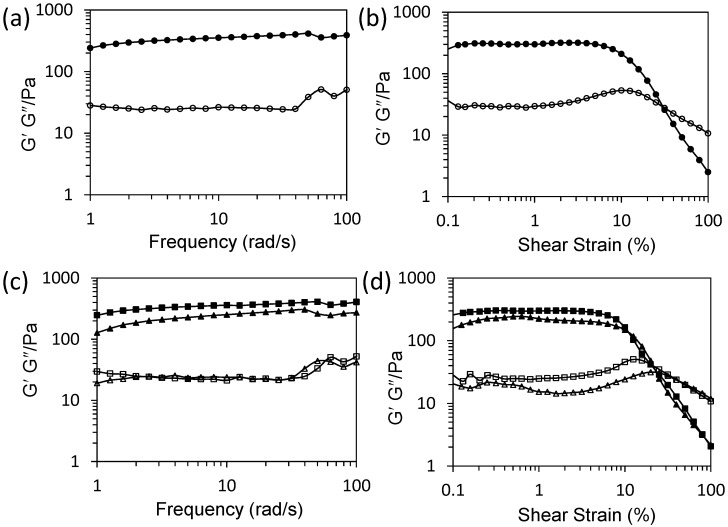
Rheometry studies showing (**a**) frequency sweep and (**b**) amplitude sweep curves for an as-prepared CNC–organoclay colloidal nanocomposite hydrogel (ratio 1:0.13); values for storage *G*′ (filled circles) and loss *G*″ moduli (open circles); (**c**) and (**d**) show frequency and amplitude sweep profiles, respectively, for a CNC–organoclay nanocomposite hydrogel (ratio 1:0.13) after exposure to gaseous ammonia (*G*′, filled triangles; *G*″, open triangles) and carbon dioxide (*G*′, filled squares; *G*″, open squares).

To probe the interactions between the exfoliated organoclay particles and CNCs, we characterized the colloidal nanocomposite hydrogels prepared at a CNC:organoclay weight ratio of 1:0.13 using zeta potential, powder X-ray diffraction (PXRD), and rheometry techniques. Zeta potential studies on the CNCs, exfoliated organoclay sheets, and CNC–organoclay nanocomposite hydrogel gave values of −43 mV, +20 mV, and −16 mV, respectively (Supporting Information, Figure S3). The significant decrease in the overall surface charge of the CNCs in the hydrogels was consistent with strong electrostatic interactions between the cationic aminopropyl-functionalities associated with the magnesium phyllosilicate framework and sulfonic functional groups of CNCs. Low-angle PXRD studies of the as-synthesized aminopropyl-functionalized magnesium phyllosilicate showed a broad reflection at 2θ = 5.9°, which was attributed to an interlayer spacing (*d*_001_) of 1.5 nm, consistent with covalently anchored aminopropyl-functionalities present in the interlamellar regions [22]. In contrast, CNC–organoclay nanocomposite hydrogel samples displayed no reflections below 2θ = 10°, confirming that the organoclay sheets remained exfoliated within the hydrogel matrix (Supporting Information, Figure S4a). High-angle PXRD pattern showed that characteristic peaks associated with CNC nanoparticles were retained at 2θ = 12.6° and 22.6° (Supporting Information, Figure S4b). Frequency sweep experiments showed a linear viscoelastic region where parallel storage (*G*′ = 240 Pa) remained higher than loss (*G*″ = 25 Pa) moduli, which is a typical characteristic for solid-like viscoelastic hydrogels (Figure 2a). Oscillatory amplitude sweeps at a constant frequency of 1 Hz of hydrogels showed a linear viscoelastic region with parallel *G*′ (290 Pa) and *G*″ (30 Pa) moduli up to a shear strain of 10%. Two crossover points observed above 10% shear strain were assigned to deformation of the hydrogel into a quasi-liquid state, indicating that the colloidal nanocomposite hydrogel exhibited responsive properties (Figure 2b).

We also investigated the gas-sensing properties of the colloidal nanocomposite hydrogels. For this, a hybrid hydrogel (CNC:organoclay, 1:0.13) was carefully exposed to ammonia gas to increase the pH of the bulk hydrogel from 9.4 to 10.4. Interestingly, the inversion tests carried out after change in the pH showed gravity-induced flow of the viscous hydrogel. Significantly, exposing the above viscous fluids to carbon dioxide gas lowered the pH value of the bulk hydrogel back to 9.4, which re-instigated cross-linking of the organic and inorganic building blocks to yield a self-supported hydrogel. Corresponding frequency sweep measurements on the ammonia-treated colloidal nanocomposite hydrogels at pH 10.4 showed a decrease in *G*′ values to 127 Pa, whilst lowering the pH back to 9.4 using gaseous carbon dioxide increased the *G*′ value to 240 Pa (Figure 2c), consistent with a decrease and restoration of the solid-like viscoelastic behavior of the hydrogel, respectively. In contrast, loss moduli (*G*″) values of the as-prepared hydrogel and hydrogels prepared at pH 10.4 or 9.4 remained unchanged. The oscillatory amplitude sweep experiments were also consistent with above observations (Figure 2d). Ammonia- and carbon dioxide-treated colloidal nanocomposite hydrogels maintained linear viscoelastic regions up to a shear strain of 10%, above which the samples became deformed.

**Figure 3:**
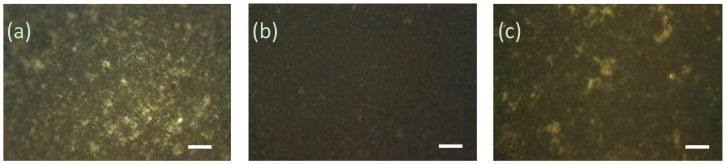
Cross-polarized microscopy images of (**a**) as-prepared (**b**) ammonia- and (**c**) carbon dioxide-treated CNC–organoclay colloidal nanocomposite hydrogels, scale bar = 100 μm.

We also employed cross-polarized (CP) optical microscopy to investigate the effect of gas-induced reassembly and disassembly of CNC–organoclay colloidal nanocomposite hydrogel networks (Figure 3). CP microscopy images of the as-prepared CNC:organoclay nanocomposite hydrogels showed birefringence, indicating the presence of anisotropic CNC–organoclay domains dispersed within an isotropic phase. Exposure to ammonia gas caused significant reduction in the birefringence of the hybrid gels, consistent with an increase in the isotropic phase due to loss of the interactions between the CNC and organoclay particles. Notably, birefringence was regained when the above samples were carefully exposed to the carbon dioxide gas, confirming that the non-covalent interactions between the organic and inorganic particles were restored.

Taken together, the above observations suggest that pH-dependent electrostatic interactions between the CNC and exfoliated organoclay sheets were primarily responsible for the formation of the self-supported hydrogels. Gas-induced disassembly and reconstruction of the CNC–organoclay nanocomposite hydrogels was attributed to deprotonation and re-protonation of aminopropyl-functionalities (pKa of primary amine ~10.5) associated with the organoclay sheets. As a consequence, environmentally induced changes in pH are able to alter the columbic interactions between the CNC and organoclay layers, and, therefore, strongly influence the mechanical and optical properties of the colloidal nanocomposite hydrogels.

The potential use of CNC–organoclay colloidal nanocomposite hydrogels for the encapsulation and controlled release of functional small molecules such as the anti-inflammatory drug, ibuprofen, was investigated. Encapsulation of the drug was carried out by mixing an aqueous solution of ibuprofen (10 wt %) with a CNC sol (6 wt %), followed by addition of an aqueous dispersion of the exfoliated organoclay sheets. The resulting mixture produced a self-supported hydrogel with a CNC:organoclay:ibuprofen weight ratio of 1:0.13:1.6. Frequency sweeps revealed a linear viscoelastic region in which the parallel storage moduli were higher than the loss moduli. However, the storage moduli (*G*′ = 121 Pa) and loss moduli (*G*″ = 13.8) values were significantly lower than that of parent CNC–organoclay hybrid hydrogel (Supporting Information, Figure S5). Oscillatory amplitude sweeps at a constant frequency of 1 Hz revealed that *G*′ and *G*″ remained parallel and deformed above 10% shear strain (Supporting Information, Figure S6). The marked decrease in solid-like viscoelastic properties of the drug-loaded hybrid hydrogel was attributed to charge screening of the columbic interactions between the CNC and exfoliated clay layers due to the presence of the anionic drug molecules.

**Figure 4:**
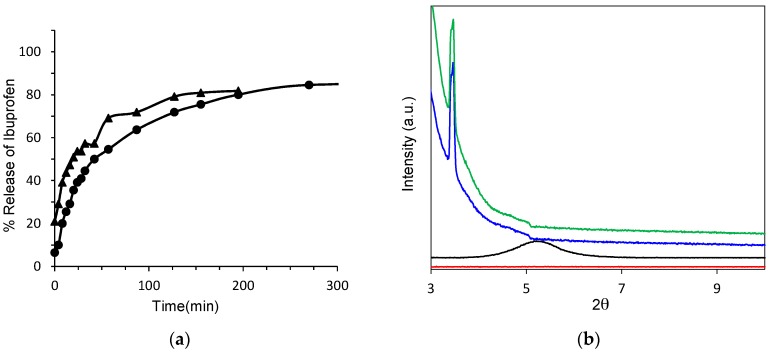
(**a**) Ibuprofen release profiles from organoclay–ibuprofen nanocomposite pellet (triangles) and CNC–organoclay–ibuprofen colloidal nanocomposite hydrogels (circles); (**b**) powder X-Ray diffraction (PXRD) pattern of CNC–organoclay nanocomposite hydrogel (red), as-synthesized organoclay (black), organoclay–ibuprofen nanocomposite (blue), and CNC–organoclay–ibuprofen colloidal nanocomposite hydrogel (green).

The concentration of ibuprofen released over time from the colloidal nanocomposite CNC–organoclay hydrogel was determined by using UV–vis spectroscopy. As a control sample, an ibuprofen–organoclay composite was prepared by adding an aqueous solution of ibuprofen to a freshly exfoliated suspension of organoclay. This allowed spontaneous restacking of the exfoliated sheets in the presence of the drug molecules. The resulting precipitates were isolated, dried, and pressed into pellet for the release studies. Both the ibuprofen-loaded colloidal nanocomposite hydrogel and organoclay–ibuprofen control nanocomposite showed a steady release of the drug molecules over a period of 6 h. Comparison of the initial release profiles obtained within an hour indicated that the extraction of ibuprofen molecules from the drug-loaded CNC–organoclay hydrogel was approximately 10% slower than that from the ibuprofen–organoclay nanocomposite pellet (Figure 4a). To further elucidate the origin of these differences, structural investigations on the nature of the ibuprofen–organoclay nanocomposite and CNC–organoclay–ibuprofen hydrogel were undertaken by using PXRD studies (Figure 4b). PXRD profiles recorded from the nanocomposite indicated that the exfoliated organoclay sheets were spontaneously restacked into a mesolamellar bulk phase in the presence of the drug molecules. As a result, the interlamellar spacing for the parent organoclay was increased from 1.5 nm (2θ = 5.9°) to 2.3 nm (2θ = 3.8°), which suggested that ibuprofen molecules were intercalated within the interlayer regions of the reconstituted organoclay. Significantly, PXRD patterns of the ibuprofen-containing CNC–organoclay hydrogels also exhibited a low-angle reflection at 2θ = 3.8° corresponding to an interlayer distance of 2.3 nm, indicating that the colloidal nanocomposite hydrogels comprised mesolamellar domains containing intercalated drug molecules [22]. The slower displacement of the drug molecules observed from the nanocomposite hydrogels could be therefore attributed to a combination of charge-mediated and diffusion-limited processes associated with interactions between the aminopropyl side chains of the organoclay sheets and carboxylic acid moieties of ibuprofen, and physical immobilization of the drug molecules within the interconnected CNC network, respectively.

## 3. Conclusions

In summary, we have demonstrated a simple methodology for construction of colloidal nanocomposite hydrogels by using aqueous sols of 1D cellulose nanocrystals and 2D delaminated sheets of aminopropyl-functionalized magnesium phyllosilicate clay. The results indicate that electrostatic interactions between the negatively charged cellulose nanocrystals and polycationic exfoliated sheets of the organoclay spontaneously generate a 3D interconnected matrix that encapsulates water molecules to yield self-supported hydrogels. We also show the colloidal CNC–organoclay nanocomposite hydrogels are responsive to external stimuli such that gas-induced changes in pH-triggered reversible changes in viscoelastic properties. Moreover, inclusion of ibuprofen in the colloidal nanocomposite hydrogels facilitates reassembly of the cationic organoclay sheets to produce mesolamellar domains comprising intercalated drug molecules. As a consequence, the hydrogel matrices show slower drug release rates. Thus, the CNC–organoclay colloidal nanocomposite hydrogels should offer an excellent opportunity to fabricate new types of stimuli-responsive hydrogels that are bioactive and biocompatible, and of potential use in applications such as wound dressing and consumer care products.

## 4. Experimental Section

### 4.1. Materials and Methods

All chemicals were obtained from Sigma-Aldrich (St. Louis, MO, USA) and used without purification. Rheology experiments were performed by using Malvern Kinexus Pro Rheometer (Malvern Instruments Ltd, Malvern, UK) equipped with parallel plate (diameter of 20 mm). Hydrogel samples aged for 1 day were added to the base plate to minimize shear. The top plate was then lowered to a set gap width of 150 µm, and the normal force was then measured; once the normal force reached an equilibrium, the measurements were performed at room temperature. CNC–organoclay hydrogel samples obtained from different batches showed similar rheological behavior and the data were within ±10% error. We ascribe the differences to changes in hydration levels and shear-induced effects during sample loading. Cross-polarized optical microscopy images were obtained by using Leica DMI3000 B manual inverted fluorescence microscope (Leica Microsystems Ltd., UK). Transmission electron microscopy analysis was undertaken on JEOL1400 operating (JEOL Ltd., Tokyo, Japan) at 120 keV in bright field mode. Energy dispersive X-ray analysis (EDX) analysis was performed by using Oxford Instrument Aztec microanalysis SDD detector (Oxford Instruments, Abingdon, UK) attached to JEOL 2100F-STEM microscope (JEOL Ltd.). CNC samples were prepared by mounting 5 µL of the 1 wt % CNC dispersions onto carbon-coated grids and left to dry at room temperature. Negative staining was carried out using an aqueous solution of 1% uranyl acetate. Delaminated sheets of organoclay were imaged by mounting freshly exfoliated dispersion of organoclay (0.01 mg/mL). TEM micrographs of hydrogel samples were obtained by dispersing 5–10 μL of gel samples in 500 μL distilled water prior to mounting onto carbon-coated grids. Zeta-potential measurements for aqueous dispersions of CNCs (1 wt %), freshly exfoliated organoclay (1 mg/mL), and hydrogels (20 μL of gel dispersed in 5 mL distilled water) were performed using Malvern Zetasizer Nano ZS (Malvern Instruments Ltd., Malvern, UK). Powder diffraction patterns were obtained by using Bruker Advanced D8 powder diffractometer (Bruker Corporation, Billerica, MA, USA). Controlled release experiments were performed by using Lambda-35 Perkin-Elmer UV–vis spectrophotometer (PerkinElmer Inc., Waltham, MA, USA).

### 4.2. Synthesis and Exfoliation of Aminopropyl-Functionalized Magnesium Phyllosilicate (Organoclay)

Magnesium chloride hexahydrate (0.84 g) was dissolved in 20 g ethanol. To this solution, 1.3 mL of 3-aminopropyltriethoxysilane was added dropwise with continuous stirring. The white slurry obtained after 5 min was stirred overnight and the precipitate was isolated by centrifugation, washed with ethanol (50 mL), and dried at 40 °C. A stable clear aqueous dispersions containing exfoliated organoclay layers (1–10 wt %) were prepared by dispersing desired amounts of organoclay powders in distilled water followed by ultrasonication for 5 min.

### 4.3. Synthesis of Crystalline Nanocellulose (CNC)

Fibrous cellulose powder (10.0 g, Whatman–CF11, Sigma-Aldrich Co.) was hydrolyzed by sulfuric acid (87.5 mL, 64%) for 40 min at 45 °C with continuous stirring. The hydrolysis was quenched by adding a large amount of water (500 mL) to the reaction mixture. The resulting mixture was cooled to room temperature 25 °C and centrifuged (4000 rpm) for 10 min at room temperature. The supernatant was decanted. Distilled water (500 mL) was added to the precipitate and the mixture was then stirred vigorously to form a new suspension. This centrifugation process was repeated three times. The newly generated suspension was dialyzed with an ultrafiltration membrane (30,000 molecular weight cutoff) until the pH of the suspension reached a constant value (pH ~ 6). Finally, the suspension was collected into glass vessel, then evaporated at 40 °C to obtain the desired concentration of CNC.

### 4.4. Fabrication of Colloidal CNC–Organoclay Nanocomposite Hydrogels

Typically, nanocrystalline cellulose-containing nanocomposite hydrogels were prepared by adding an aqueous solution of exfoliated organoclay at low concentrations (1–10 wt %) to a colloidal sol of CNC with low concentration (3 wt %). The mixture was allowed to stand for several minutes to obtain a self-supported CNC–organoclay hybrid hydrogel.

### 4.5. Synthesis of CNC–Organoclay–Ibuprofen Nanocomposite Hydrogels

Typically, 0.1 mL of a colloidal sol of CNC at 6 wt % concentration was added to 0.1 mL of an aqueous ibuprofen (10 wt %) solution with stirring. Freshly exfoliated organoclay (5 wt %, 0.016 mL) dispersions were added and the reaction mixture was allowed to stand to obtain the CNC–organoclay–ibuprofen hydrogel.

### 4.6. Synthesis of Organoclay–Ibuprofen Composites

Ibuprofen solution (0.1 mg/mL) was added to a freshly prepared exfoliated dispersion of organoclay (0.05 mg/mL). The resulting precipitates were centrifuged, dried, and pressed into pellets for drug-release experiments.

### 4.7. Controlled Release Studies

Organoclay–ibuprofen pellets or CNC–organoclay–ibuprofen hydrogels were immersed in a known volume of distilled water. Aliquots were removed at regular time intervals and time-dependent release of ibuprofen was measured by monitoring changes in the intensity of the absorption peak at 264 nm using UV–vis spectroscopy. Release profiles were plotted by averaging absorption values obtained over three runs.

## Supplementary Materials

The following are available online at www.mdpi.com/2310-2861/3/1/11/s1. Supporting figures of TEM, zeta potential measurements, powder X-ray diffraction patterns, and rheology data.
