# Supplementary material for: Self-Assembly of Colloidal Nanocomposite Hydrogels Using 1D Cellulose Nanocrystals and 2D Exfoliated Organoclay Layers
Source: Gels. 2017 Mar 17;3(1):11. doi: 10.3390/gels3010011 (PMC6318600; doi:10.3390/gels3010011)
Supplement: Supplementary file 1 [file gels-03-00011-s001.pdf]

Supporting Figures: Self-Assembly of Colloidal Nanocomposite Hydrogels Using 1D Cellulose Nanocrystals and 2D Exfoliated Organoclay Layers

T. Okamoto et al.

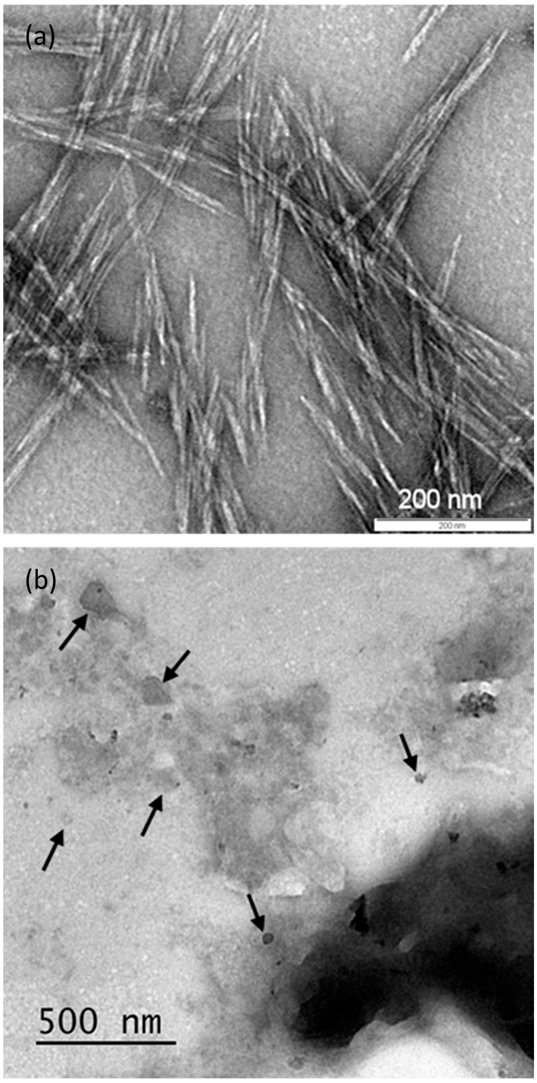

Figure S1: TEM images showing (**a**) uranyl acetate-stained cellulose nanocrystals (CNCs) and (**b**) exfoliated sheets of aminopropyl-functionalized magnesium phyllosilicate clay, indicated by arrows.

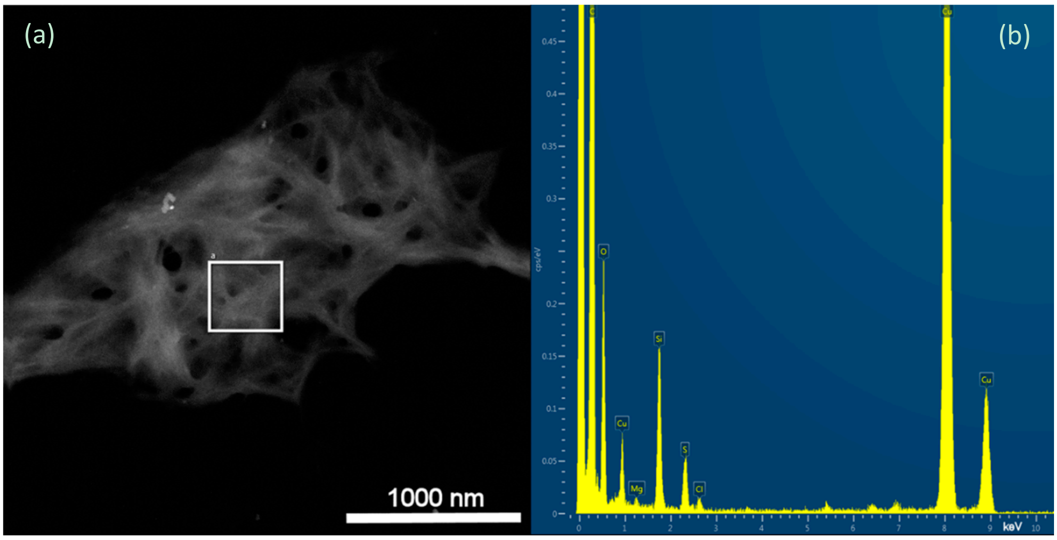

Figure S2: (**a**) High-angle annular dark-field STEM image of CNC–organoclay hydrogel sample; square box shows the area selected for EDX analysis; (**b**) EDX analysis of a corresponding gel sample.

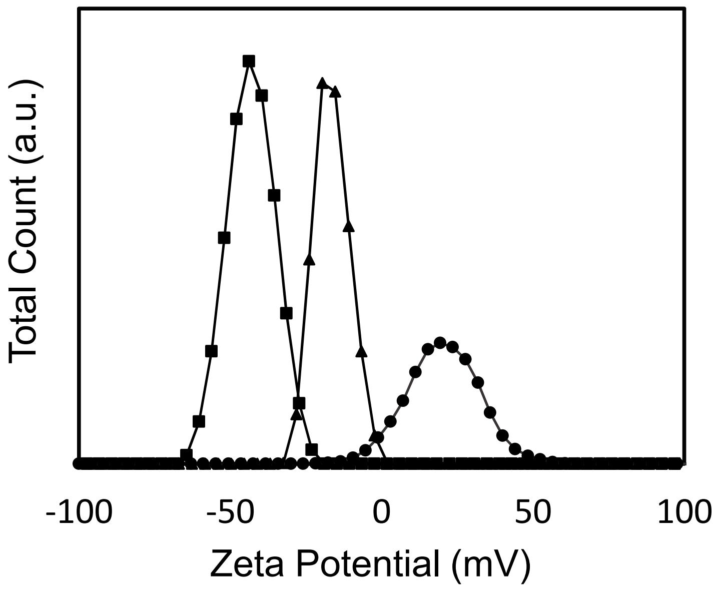

Figure S3: Zeta-potential profiles for CNCs (squares), freshly exfoliated organoclay sheets (circles), and a CNC–organoclay hydrogel dispersion (triangles).

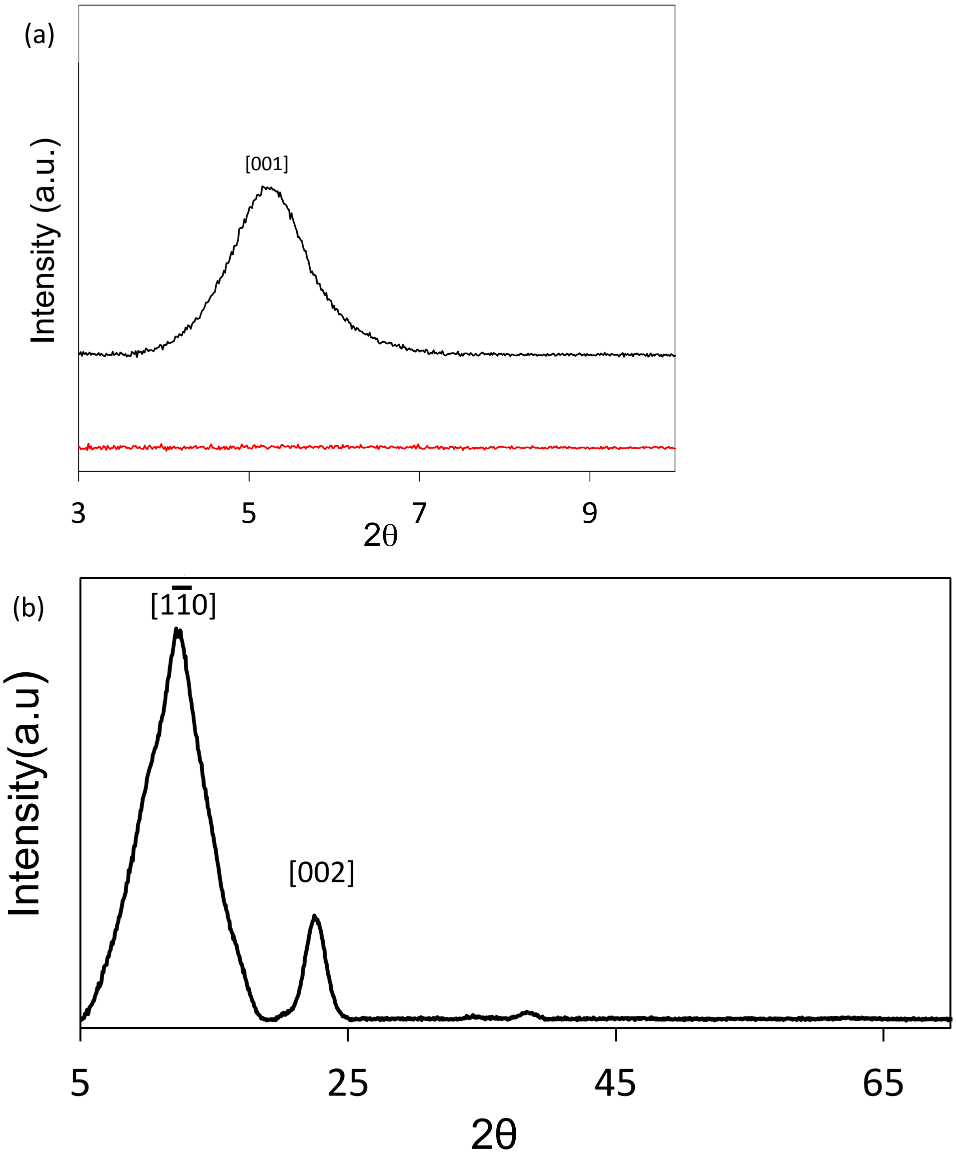

Figure S4: (**a**) Low-angle PXRD patterns of as-synthesized organoclay (black) and CNC–organoclay nanocomposite hydrogel (red), (**b**) high-angle PXRD pattern of CNC–organoclay nanocomposite hydrogels showing [1$\bar{1}$0] and [002] reflections associated with CNCs.

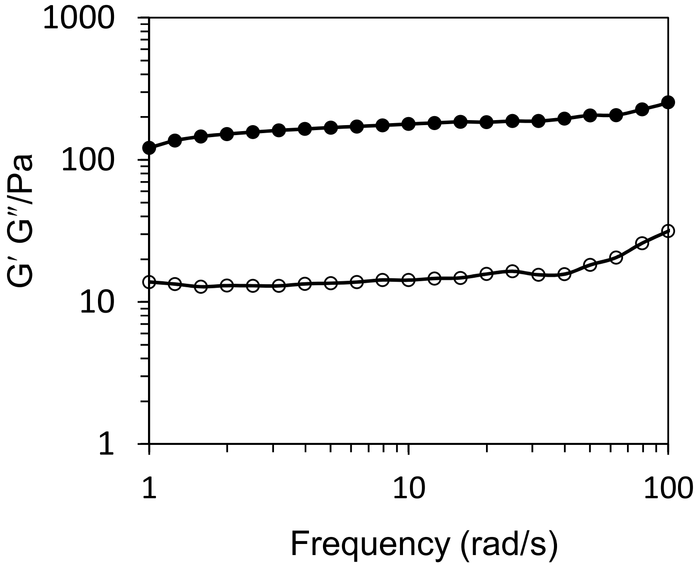

Figure S5: Frequency sweep profile showing storage G′ (filled circles) and loss G″ moduli (open circles) of CNC–organoclay–ibuprofen nanocomposite hydrogel.

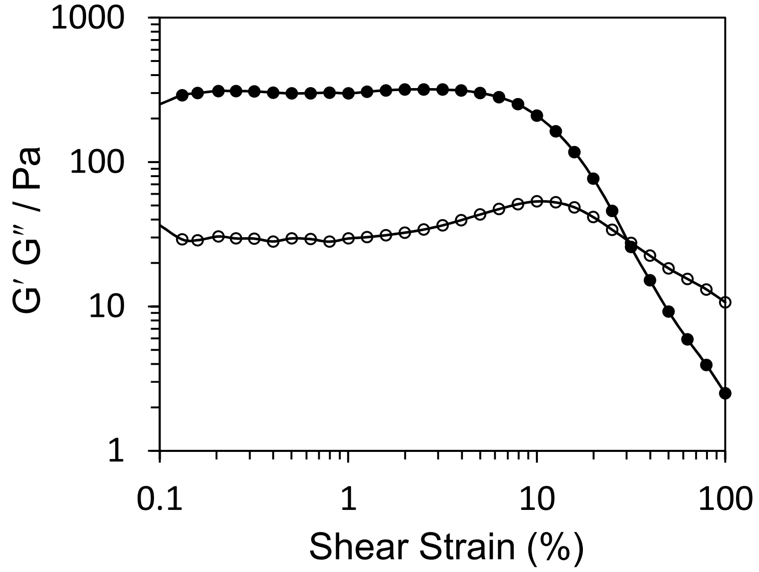

Figure S6: Oscillatory amplitude sweep curves showing storage G′ (filled circles) and loss G″ moduli (open circles) at a constant frequency of 1 Hz for CNC–organoclay–ibuprofen hybrid hydrogel.
